# Obstetric complications and mother’s age at delivery are predictors of eating disorder symptoms among Health Science college students

**DOI:** 10.1590/S1679-45082015AO3366

**Published:** 2015

**Authors:** Mara Cristina Lofrano-Prado, Wagner Luiz do Prado, Mauro Virgilio Gomes de Barros, Thiago Ricardo dos Santos Tenório, Sandra Lopes de Souza

**Affiliations:** 1 Universidade Federal de Pernambuco, Recife, PE, Brazil.; 2 Universidade Federal de São Paulo, Santos, SP, Brazil.; 3 Universidade de Pernambuco, Recife, PE, Brazil.

**Keywords:** Anorexia nervosa, Bulimia nervosa, Eating disorders, Pregnancy complications, Students

## Abstract

**Objective:**

To identify the association between perinatal/neonatal factors and symptoms of eating disorders among college students.

**Methods:**

Four hundred and eight college students (283 women), aged 18 to 23 years old, enrolled in the first semester of a Bachelor of Health Science degree program were included in the sample. Eating disorder symptoms and body image dissatisfaction were assessed with the Eating Attitudes Test and Bulimic Investigatory Test of Edinburgh. Information regarding birth weight, breastfeeding, obstetric complications, mother’s age at delivery, type of delivery, and birth order were self-reported by the volunteers after consulting their parents. Association between perinatal and neonatal factors and symptoms of anorexia nervosa and bulimia nervosa were assessed by binary logistic regression adjusted for sex, age, and body mass index.

**Results:**

The likelihood of presenting with symptoms of anorexia nervosa was 0.5 time lower for those students born from the oldest mothers (odds ratio – OR=0.37; 95% confidence interval – 95%CI: 0.17-0.83). Relative to bulimia nervosa, the risk was higher among students who reported obstetric complications (OR=2.62; 95%CI: 1.03-6.67).

**Conclusion:**

We observed the association between perinatal and neonatal factors with symptoms of eating disorders in college students.

## INTRODUCTION

In the late 1980s, Barker et al.^(1)^ were pioneers in showing that low birth weight was associated with increased risk of death in adulthood, and since then it has been hypothesized that intrauterine environment, as well as the early development period are responsible for adaptive responses that could predict the risk for some diseases in later life. Some years later, Hales and Barker^(2)^ developed the “thrifty phenotype hypothesis”, which currently is the essence of the developmental origins of health and disease (DOHaD) hypothesis.^(3,4)^

It is well established that perinatal and neonatal environment may predict the likelihood of psychiatric disorders,^(5-7)^ cardiovascular, and metabolic diseases,^(3,8)^ and are involved in the etiopathogenesis of obesity.^(9)^ In this way, it is feasible to hypothesize that they may occur in the etiology of eating disorders (ED).

Eating disorders are psychiatric disturbances of complex etiology, and although many empirical data are available about the possible role of a variety of putative risk factors, almost nothing is known about the real pathways to ED. Nowadays, there is some evidence that neonatal environment may be involved in the etiopathogenesis of anorexia nervosa and bulimia nervosa.^(10-15)^

According to the Diagnostic and Statistical Manual of Mental Disorders IV (DSM-IV),^(16)^ anorexia nervosa is characterized by an intense fear of weight gain or becoming fat, body image distortion, and amenorrhea; bulimia nervosa is categorized by recurrent episodes of binge eating, sense of lack of control over eating during the episode, recurrent inappropriate compensatory behavior in order to gain weight, such as self-induced vomiting, misuse of laxatives, diuretics, enemas, or other medications.

Despite the relevance of ED to Public Health, only few studies addressed the interplay between perinatal/neonatal factors and ED, and were carried out on clinical populations.^(9-11)^ The current literature neglects individuals vulnerable to developing ED,^(17)^ and it is well known that college is a critical period for onset of ED.^(18)^

## OBJECTIVE

To identify the association between perinatal/neonatal factors and symptoms of eating disorders among college students.

## METHODS

### Participants

This cross-sectional study was performed with 408 college students enrolled in the first semester of a Bachelor of Health Sciences degree program (Medicine, Nutrition, Physical Education, Physical Therapy, Dentistry, Nursing, and Occupational Therapy) at public universities from Recife (PE), Northeastern region of Brazil. The subjects were 125 men and 283 women aged between 18 to 23 years, and enrolled in the first year of college. The exclusion criteria were to be enrolled in other Health Science classes or semesters (to avoid previous knowledge about the instruments), pregnancy, and adoption (infeasibility of having pre- and neonatal information). This study was performed in accordance with the principles of the Declaration of Helsinki and was formally approved by the Ethical Committee of the *Universidade Federal de Pernambuco *CAAE: 0143.0.172.000-10. Informed Consent was obtained from all subjects.

The sample size was estimated with a 5% margin of error (95% confidence interval − 95%CI). A design effect of 1.5 and 50% expected prevalence of ED symptoms was based on previously studies.^(19-22)^ The estimated sample size was sufficient to detect significant odds ratio values ≥1.8, with a power of 80% and a 95%CI.

### Measurements

At the end of a regular class period, subjects were invited to participate as volunteers after an explanation of the objectives and procedures of the study. Those who agreed to participate were submitted to the assessment procedures including questionnaires and anthropometric measurements. Information regarding birth weight, breastfeeding, obstetric complications, mother’s age at delivery, type of delivery, and birth order were self-reported by the volunteers after consulting with their parents.

Standing weight and height were measured on scales (Filizola, São Paulo (SP), Brazil) to the nearest 0.1kg and 0.5cm, respectively, according to Jackson and Pollock.^(23)^ Body mass index (BMI) was calculated by dividing body weight (kg) by height squared (m^(2)^).

The questionnaires were applied by a psychologist in a separate and quiet room, following the instrument’s procedures.

### Eating Attitudes Test

Translated and validated for the Brazilian population by Nunes et al.,^(24)^ the Eating Attitudes Test (EAT-26) is a self-reported instrument employed in the evaluation and identification of abnormal eating patterns. The instrument comprises 26 items, with 6 reply options: “always” corresponds to 3 points, “very frequently” corresponds to 2 points, “frequently” corresponds to 1 point, “sometimes/rarely/never” corresponds to zero point. Scores above 20 points were classified as likelihood of showing abnormal eating behavior and risk of developing anorexia nervosa.^(25)^

### Bulimic Investigation Test of Edinburgh

Translated and validated for the Brazilian population by Cordás and Hochgraf,^(26)^ the Bulimic Investigatory Test of Edinburgh (BITE) is a 33-item self-reported measurement designed to identify symptoms of bulimia or binge eating. BITE consists of two subscales: the symptoms and the severity scale. Scores of the symptoms scale are subdivided into three categories: high scores (higher than 20); medium scores (between 10 and 19); and low scores (below 10). The other scale measures the severity of bingeing and purging behavior, defined by frequency.^(27)^

In the end, variables were dichotomized as follows: presence or absence of symptoms of ED; birth weight higher or lower than 2,500g; breastfeeding or not; obstetric complications or not; mother’s age at delivery higher or lower than 25 years of age; cesarean section or vaginal delivery; and first in birth order at delivery or not.

### Statistical analysis

All data were analyzed by Statistical Package for Social Science (SPSS) version 10 for Windows. The Komolgorov-Smirnov test was performed to verify the normality of the data. Since the distribution was not normal, data was expressed by median, minimum and maximum values. Comparisons between groups were verified by the Mann-Whitney U test. For categorical variables, data are presented as absolute and relative frequencies, and differences between groups were analyzed by χ^2^ test. Associations between perinatal/neonatal factors and symptoms of anorexia nervosa and bulimia nervosa were assessed by binary logistic regression. Results are shown as unadjusted and adjusted odds ratio values, and 95%CI. Significance was set at p<0.05.

## RESULTS

The sample was composed of 408 college students, 283 females (69.4%). A total of 115 (28.2%; 95%CI: 23.9%-32.7%) students presented with symptoms of ED. Table 1 shows that students with symptoms of ED were heavier (p<0.001) and had a higher BMI (p<0.001) than those without symptoms.

**Table 1:** Anthropometric data and perinatal/neonatal factors in college students presenting or not eating disorders symptoms

Variables	ED symptoms (n=115)		No ED symptoms (n=293)	
	Median	Min-Max	Median	Min-Max
Age (years)	19.10	18.00-23.31	19.32	18.00-23.86
Height (m)	1.64	1.46-1.86	1.66	1.48-1.92
Body mass (kg)	63.30	45.00-111.50	58.00*	40.00-105.00
BMI (kg/m^2^)	23.66	17.49-37.82	21.22*	15.65-33.89
Birth weight (g)	3.40	2.00-4.80	3.41	1.80-4.96
Mother’s age at delivery (years)	27	16-45	27	14-41
Breastfeeding (months)	6.00	1.00-48.00	6.00	1.00-60.00

*Versus ED symptoms. p<0.001. Min: minimum value; max: maximum value; BMI: body mass index; ED: eating disorders.

Out of all students with symptoms of ED, 61.7% (95%CI: 51.6-71.1) the mother’s age at delivery was ≥25 years, 11.4% (95%CI: 6.3-18.6) presented with obstetric complications, 2.9% (95%CI: 0.7-7.6) had not been breastfed, 60.6% (95%CI: 50.5-70.1) were delivered by cesarean section, and 46.8% (95%CI: 36.9-56.9%) were first in birth order. The most prevalent obstetric complications were pregnancy-related diseases, such as bleeding, preeclampsia, diabetes, threatened miscarriages, and anemia, followed by premature rupture of the membranes, oxygen and ventilation required at birth, umbilical cord knots or loops, and others not specified.

Multivariate binary logistic regression revealed that the likelihood of presenting symptoms of anorexia nervosa was 0.5 times lower for those students born from the oldest mothers (≥25 year-old) (Table 2). Relative to bulimia nervosa, the risk is 2.6 times higher among students who reported obstetric complications (Table 3). For birth weight, breastfeeding, type of delivery, and birth order, no associations were found.

**Table 2:** Odds ratio for anorexia nervosa according to neonatal characteristics in college students

Variables	Crude OR (95%CI)	Wald	p value	Adjusted OR (95%CI)*	Wald	p value
Low birth weight (<2,500g)	0.61 (0.14-2.71)	0.41	0.52	0.59 (0.11-3.33)	0.36	0.55
No breastfeeding	1.16 (0.33-4.12)	0.05	0.82	1.39 (0.34-5.71)	0.20	0.65
Obstetric complications	2.04 (0.87-4.77)	2.68	0.10	2.32 (0.83-6.50)	2.55	0.11
Mother’s age at delivery ≥25 years	0.50 (0.26-0.96)	4.38	0.04	0.37 (0.17-0.83)	5.85	0.02
Cesarean delivery	1.14 (0.59-2.18)	0.15	0.70	1.41 (0.66-3.02)	0.79	0.37
Not first in the birth order	1.07 (0.55-2.08)	0.04	0.85	1.86 (0.86-4.02)	2.51	0.11

*Adjusted for sex, age, and body mass index. 95%CI: 95% confidence interval; OR: odds ratio.

**Table 3:** Odds ratio for bulimia nervosa according to the neonatal characteristics in college students

Variables	Crude OR (95%CI)	Wald	p value	Adjusted OR (95%CI)*	Wald	p value
Low birth weight (<2,500g)	0.46 (0.13-1.57)	1.55	0.21	0.36 (0.72-1.83)	1.51	0.22
No breastfeeding	0.56 (0.16-1.95)	0.84	0.36	0.81 (0.19-3.37)	0.09	0.77
Obstetric complications	1.28 (0.60-2.77)	0.39	0.53	2.62 (1.03-6.67)	4.09	0.04
Mother’s age at delivery ≥25 years	0.80 (0.47-1.35)	0.72	0.40	0.67 (0.34-1.34)	1.28	0.26
Cesarean delivery	1.13 (0.67-1.91)	0.22	0.64	0.94 (0.49-1.79)	0.03	0.85
Not first in the birth order	1.10 (0.66-1.84)	0.15	0.70	1.51 (0.79-2.89)	1.56	0.21

*Adjusted for sex, age, and actual body mass index. 95%CI: 95% confidence interval; OR: odds ratio.

## DISCUSSION

This is the first study conducted with a non-clinical sample, comprised of freshman Health Sciences students aiming to explore the interplay between perinatal/neonatal factors and symptoms of ED. Since some studies indicated that this population is more vulnerable to developing ED,^(17,28)^ the present investigation adds relevant data to current knowledge regarding the possible agents involved in the etiology of ED. Thus, the main findings are: (1) obstetric complications are positively associated with symptoms of bulimia nervosa, and (2) a mother’s age lower than 25 years at delivery is a risk factor for symptoms of anorexia nervosa.

Our data showed that symptoms of bulimia nervosa are 2-fold higher among students with obstetric complications than peers not presenting them. In agreement with our results, Favaro et al.^(12)^ verified the same pattern in females with diagnosis of anorexia nervosa. Additionally, Kelly et al.^(29)^ demonstrated that obstetric complications could be involved in the onset of schizophrenia.

Since perinatal environment and early development period may induce long-lasting changes in brain structure and plasticity,^(30)^ as well as in organogenesis and the epigenetic profile in newborns,^(31)^ the possible mechanisms underlying the deleterious effects of obstetric complications may be mediated by hypoxia, which causes impairment of the neurological development of the newborn.^(32)^ Moreover, malnutrition during pregnancy and in the postnatal period seem to regulate energy intake and appetite in later life.^(33)^ It is important to note that different types of obstetric complications have diverse implications for the risk of developing psychiatric illness.^(34)^

A surprising result from the present study is the lower symptoms of anorexia nervosa in those students born from mothers older than 25 years of age; unfortunately, there are no data available regarding this topic to help us to draw any hypothesis for this issue. However this intriguing fact should be investigated in future studies to better elucidate this possible association.

Our study indicates that birth weight, breastfeeding, type of delivery, and birth order do not influence the likelihood for ED symptomatology. In accordance with our data, Nicholls and Viner,^(35)^ in a prospective national birth cohort, did not identify a dose-response relation between low birth weight and risk of anorexia nervosa. Favaro et al.,^(12)^ in a longitudinal cohort study in a clinical population with anorexia and bulimia nervosa, found no association with low birth weight and birth order as a risk factor for developing ED.

The present study has some limitations that should be considered. The self-reported measurement of perinatal and neonatal periods may have been influenced the assessed outcomes. Due to the large range of obstetric complications reported in the present study, we were not able to analyze the isolated association of each obstetric complication with the symptoms of ED. Because of these limitations, the interpretation of the data needs to be done with caution. In this way, future studies conducted with a large sample size, analyzing the single and clustered neonatal and perinatal factors could shed light and enhance our understanding regarding this relevant topic in Public Health.

The strengths of the present study are the volunteer’s characteristics: such as freshman Health Sciences students, the inclusion of males in the sample, and the adjustment of the analysis for sex and BMI.

## CONCLUSION

Perinatal and neonatal factors are important in the pathogenesis of eating disorders among college students. Accordingly, careful surveillance is needed in order to avoid the increase in the incidence of eating disorders in this population. We suggest that universities pay attention to this problem, provide psychological support for students as from the beginning of college; and give information on prevention of eating disorders during orientation classes for freshman Health Sciences students, to avoid future complications in the next generation of health professionals. Further studies should focus on the underlying mechanisms that link neonatal factors to eating disorders.
