# Supplementary material for: Obstetric complications and mother’s age at delivery are predictors of eating disorder symptoms among Health Science college students
Source: Einstein (Sao Paulo). 2015 Oct-Dec;13(4):525–9. doi: 10.1590/S1679-45082015AO3366 (PMC4878625; doi:10.1590/S1679-45082015AO3366)
Supplement: Translation

# Translation: Complicações obstétricas e idade materna no parto são preditores de sintomas de transtornos alimentares em estudantes universitários da área da Saúde

**Authors:** Mara Cristina Lofrano-Prado, Wagner Luiz do Prado, Mauro Virgilio Gomes de Barros, Thiago Ricardo dos Santos Tenório, Sandra Lopes de Souza

**Affiliations:** 1 Universidade Federal de Pernambuco, Recife, PE, Brasil.; 2 Universidade Federal de São Paulo, Santos, SP, Brasil.; 3 Universidade de Pernambuco, Recife, PE, Brasil.

**Keywords:** Anorexia nervosa, Bulimia nervosa, Transtornos alimentares, Complicações na gravidez, Estudantes

## Abstract

**Objetivo:**

Identificar a associação entre fatores perinatais/neonatais e sintomas de transtornos alimentares entre estudantes universitários.

**Métodos:**

Quatrocentos e oito estudantes universitários (283 mulheres), com idade entre 18 e 23 anos, matriculados no primeiro semestre de cursos de Bacharelado na área das Ciências da Saúde foram incluídos na amostra. Sintomas de transtornos alimentares e de insatisfação com a imagem corporal foram avaliados por meio do *Eating Attitudes Test *e do* Bulimic Investigatoty Test of Edinburgh*. Informações sobre o peso ao nascer, aleitamento materno, complicações obstétricas, idade materna no momento do parto, tipo de parto e ordem de nascimento foram relatadas pelos voluntários após consulta com seus pais. A associação entre fatores perinatais/neonatais e sintomas de anorexia nervosa e bulimia nervosa foi avaliada por meio de regressão logística binária ajustada por sexo, idade e índice de massa corporal.

**Resultados:**

A probabilidade de apresentar sintomas de anorexia nervosa foi 0,5 vez mais baixa para os alunos nascidos de mães mais velhas (*odds ratio* – OR=0,37; intervalo de confiança de 95% – IC 95%=0,17-0,83). Em relação à bulimia nervosa, o risco foi maior entre os estudantes que relataram complicações obstétricas (OR=2,62; IC 95%=1,03-6,67).

**Conclusão:**

Observou-se associação entre fatores perinatais e neonatais com sintomas de transtornos alimentares em estudantes universitários.

## INTRODUÇÃO

No final da década de 1980, Barker et al.^(1)^ foram pioneiros em mostrar que baixo peso ao nascer estava associado a um risco aumentado de morte na vida adulta, e, desde então, existe a hipótese de que o ambiente intrauterino, assim como o período de desenvolvimento inicial, sejam responsáveis pelas respostas de adaptação que poderiam predizer o risco de surgimento de algumas doenças posteriormente. Alguns anos depois, Hales e Barker^(2)^ desenvolveram a “hipótese do fenótipo poupador”, que atualmente é a essência da teoria das origens de saúde e doença no desenvolvimento (DOHaD, *developmental origins of health and disease*).^(3,4)^

Já está bem estabelecido que os ambientes perinatal e neonatal podem predizer a probabilidade de transtornos psiquiátricos,^(5-7)^ cardiovasculares e doenças metabólicas,^(3,8)^ e que estão envolvidos na etiopatogenia da obesidade.^(9)^ Desta forma, é viável considerar que eles podem ocorrer na etiologia de transtornos alimentares (TA).

Transtornos alimentares são distúrbios psiquiátricos de etiologia complexa e, embora existam muitos dados empíricos sobre o possível papel de uma variedade de supostos fatores de risco, quase nada é conhecido sobre as reais vias de TA. Atualmente, existe alguma evidência de que o ambiente neonatal possa estar envolvido na etiopatogenia de anorexia nervosa e bulimia nervosa.^(10-15)^

Segundo o Manual Diagnóstico e Estatístico de Transtornos Mentais IV (DSM-IV),^(16)^ anorexia nervosa caracteriza-se por um intenso medo de ganhar peso ou de engordar, distorção da imagem corporal e amenorreia. Já na bulimia nervosa, ocorrem episódios recorrentes de compulsão alimentar, sentimento de falta de controle sobre a alimentação durante o episódio, comportamento compensatório inapropriado recorrente para ganhar peso, como provocação de vômitos, uso inadequado de laxantes, diuréticos, enemas ou outras medicações.

Apesar da relevância de TA para a Saúde Pública, poucos estudos abordaram a inter-relação entre fatores perinatais/neonatais e TA, e foram realizados em populações clínicas.^(9-11)^ A literatura atual negligencia os indivíduos vulneráveis ao desenvolvimento de TA,^(17)^ e sabe-se que a fase universitária é um período crítico para o aparecimento desses transtornos.^(18)^

## OBJETIVO

Identificar a associação entre fatores perinatais/neonatais e sintomas de transtornos alimentares entre estudantes universitários.

## MÉTODOS

### Participantes

Este estudo transversal foi realizado com 408 universitários matriculados no primeiro semestre de um programa de Bacharel em Ciências da Saúde (Medicina, Nutrição, Educação Física, Fisioterapia, Odontologia, Enfermagem e Terapia Ocupacional) em universidades públicas de Recife (PE), no nordeste do Brasil. Os participantes foram 125 homens e 283 mulheres com idades entre 18 e 23 anos, matriculados no primeiro ano de faculdade. Os critérios de exclusão foram estar inscrito em outros cursos ou semestres de Ciências da Saúde (para evitar conhecimento prévio dos instrumentos), gravidez e adoção (inviabilidade de obter informações pré- e neonatais). Este estudo foi conduzido segundo os princípios da Declaração de Helsinque e foi formalmente aprovado pela Comissão de Ética da Universidade Federal de Pernambuco CAAE: 0143.0.172.000-10. O Consentimento Livre e Esclarecido foi obtido de todos os sujeitos.

O tamanho da amostra foi estimado com uma margem de erro de 5% (intervalo de confiança de IC95% - IC95%). Um efeito de delineamento de 1,5 e 50% de prevalência esperada de sintomas de TA foi baseado em estudos anteriores.^(19-22)^ O tamanho de amostra estimado foi suficiente para detectar valores de *odds ratio* significantemente ≥1,8, com um poder de 80% e um IC95%.

### Medidas

Ao final de um período regular de aulas, os estudantes foram convidados a participar como voluntários após uma explicação sobre os objetivos e os procedimentos da pesquisa. Aqueles que concordaram em participar foram submetidos aos procedimentos de avaliação, que incluíam questionários e medidas antropométricas. As informações a respeito de peso no nascimento, amamentação, complicações obstétricas, idade materna na ocasião do parto, tipo de parto, e ordem de nascimento foram relatadas após consulta com seus pais.

O peso e altura de pé foram medidos em balança (Filizola, São Paulo (SP), Brasil) até o 0,1kg e 0,5cm mais próximos, respectivamente, como proposto por Jackson e Pollock.^(23)^ O índice de massa corporal (IMC) foi calculado pela divisão do peso corpóreo (kg) pelo quadrado da altura (m^2^).

Os questionários foram aplicados por um psicólogo em uma sala separada e silenciosa, seguindo os procedimentos do instrumento.

#### Eating Attitudes Test

Traduzido e validado para a população brasileira por Nunes et al.,^(24)^ o *Eating Attitudes Test *(EAT-26) é um instrumento de autorrelato usado na avaliação e identificação de padrões anormais de alimentação. O instrumento é composto por 26 itens, com 6 opções de resposta: “sempre” corresponde a 3 pontos, “muito frequentemente” corresponde a 2 pontos, “frequentemente” corresponde a 1 ponto, e “algumas vezes/raramente/nunca” corresponde a zero ponto. Os escores acima de 20 pontos foram classificados como probabilidade de demonstrar comportamento alimentar anormal e risco de desenvolver anorexia nervosa.^(25)^

#### Bulimic Investigatory Test of Edinburgh

Traduzido e validado para a população brasileira por Cordás e Hochgraf,^(26)^ o *Bulimic Investigatory Test of Edinburgh* (BITE) é uma medida de 33 itens autorrelatados, para identificar sintomas de bulimia ou compulsão alimentar. O BITE tem duas subescalas: escala de sintomas e escala de gravidade. Os escores da escala de sintomas são subdivididos em três categorias: escores altos (maiores que 20); escores médios (entre 10 e 19); e escores baixos (abaixo de 10). A outra escala mede a gravidade da alimentação compulsiva e comportamento purgativo, definido pela frequência.^(27)^

No fim, as variáveis foram divididas da seguinte forma: presença ou ausência de sintomas de TA; peso no nascimento maior ou menor que 2.500g; amamentação ou não; complicações obstétricas ou não; idade materna na ocasião do parto maior ou menor que 25 anos; cesariana ou parto normal; e primeiro na ordem de nascimento ou não.

## Análise estatística

Todos os dados foram analisados por meio do *software Statistical Package for Social Science* (SPSS), versão 10 para *Windows*. O teste de Komolgorov-Smirnov foi feito para verificar a normalidade dos dados. Já que a distribuição não foi normal, os dados foram expressos por mediana, valor mínimo e valor máximo. As comparações entre os dois grupos foram verificados pelo teste U de Mann-Whitney. Para variáveis categóricas, os dados são apresentados como frequências absoluta e relativa, e as diferenças entre os grupos foram analisadas pelo teste χ^2^. As associações entre fatores perinatais/neonatais e os sintomas de anorexia nervosa e bulimia nervosa foram avaliadas por regressão logística binária. Os resultados são mostrados como valores de *odds ratio* não ajustados e ajustados, com IC95% A significância foi estabelecida em p<0,05.

## RESULTADOS

A amostra foi composta de 408 universitários, sendo 283 do sexo feminino (69,4%). Apresentaram sintomas de TA 115 (28,2%; IC95%: 23,9%-32,7%) estudantes. A tabela 1 mostra que os estudantes com sintomas de TA tinham mais peso (p<0,001) e maior IMC (p<0,001) que aqueles sem sintomas.

**Tabela 1:** Dados antropométricos e fatores perinatais/neonatais em universitários com ou sem sintomas de transtornos alimentares

Variáveis	Sintomas de TA (n=115)		Sem sintomas de TA (n=293)	
	Mediana	Min-Max	Mediana	Min-Max
Idade (anos)	19,10	18,00-23,31	19,32	18,00-23,86
Altura (m)	1,64	1,46-1,86	1,66	1,48-1,92
Massa corporal (kg)	63,30	45,00-111,50	58,00*	40,00-105,00
IMC (kg/m^2^)	23,66	17,49-37,82	21,22*	15,65-33,89
Peso ao nascer (g)	3,40	2,00-4,80	3,41	1,80-4,96
Idade materna no parto (anos)	27	16-45	27	14-41
Amamentação (meses)	6,00	1,00-48,00	6,00	1,00-60,00

*Versus sintomas de TA. p<0,001.Min: valor mínimo; max: valor máximo; IMC: índice de massa corporal. TA: transtornos alimentares.

De todos os estudantes com sintomas de TA, 61,7% (IC95%: 51,6-71,1) registravam idade materna no parto ≥25 anos, 11,4% (IC95%: 6,3-18,6) apresentaram complicações obstétricas, 2,9% (IC95%: 0,7-7,6) não tinham sido amamentados, 60,6% (IC95%: 50,5-70,1) nasceram de cesariana e 46,8% (IC95%: 36,9-56,9) foram os primeiros em ordem de nascimento. As complicações obstétricas mais prevalentes foram doenças relacionadas à gestação, como sangramentos, pré-eclâmpsia, diabetes, ameaça de abortos espontâneos e anemia, seguidas de ruptura prematura de membranas, necessidade de oxigênio e ventilação ao nascimento, nó no cordão umbilical ou circular de cordão, e outros não especificados.

A regressão logística multivariada binária mostrou que a probabilidade de apresentar sintomas de anorexia nervosa foi 0,5 vez menor para aqueles estudantes nascidos de mães mais velhas (≥25 anos de idade) (Tabela 2). Relativo à bulimia nervosa, o risco foi 2,6 vezes maior entre estudantes que relataram complicações obstétricas (Tabela 3). Para peso ao nascer, amamentação, tipo de parto, e ordem de nascimento, nenhuma associação foi encontrada.

**Tabela 2:** *Odds ratio* para anorexia nervosa, segundo características neonatais em universitários

Variáveis	OR bruta (IC95%)	Wald	Valor de p	OR ajustada (IC95%)*	Wald	Valor de p
Baixo peso ao nascer (<2.500g)	0,61 (0,14-2,71)	0,41	0,52	0,59 (0,11-3,33)	0,36	0,55
Sem amamentação	1,16 (0,33-4,12)	0,05	0,82	1,39 (0,34-5,71)	0,20	0,65
Complicações obstétricas	2,04 (0,87-4,77)	2,68	0,10	2,32 (0,83-6,50)	2,55	0,11
Idade materna no parto ≥25 anos	0,50 (0,26-0,96)	4,38	0,04	0,37 (0,17-0,83)	5,85	0,02
Parto cesáreo	1,14 (0,59-2,18)	0,15	0,70	1,41 (0,66-3,02)	0,79	0,37
Não o primeiro na ordem de nascimento	1,07 (0,55-2,08)	0,04	0,85	1,86 (0,86-4,02)	2,51	0,11

*Ajustado para sexo, idade e índice de massa corporal. IC95%: intervalo de confiança de 95%; OR: *odds ratio*.

**Tabela 3:** *Odds ratio* para bulimia nervosa, segundo as características neonatais em universitários

Variáveis	OR bruta (IC95%)	Wald	Valor de p	OR ajustada (IC95%)*	Wald	Valor de p
Baixo peso ao nascer <2.500g)	0,46 (0,13-1,57)	1,55	0,21	0,36 (0,72-1,83)	1,51	0,22
Sem amamentação	0,56 (0,16-1,95)	0,84	0,36	0,81 (0,19-3,37)	0,09	0,77
Complicações obstétricas	1,28 (0,60-2,77)	0,39	0,53	2,62 (1,03-6,67)	4,09	0,04
Idade materna no parto ≥25 anos	0,80 (0,47-1,35)	0,72	0,40	0,67 (0,34-1,34)	1,28	0,26
Parto cesáreo	1,13 (0,67-1,91)	0,22	0,64	0,94 (0,49-1,79)	0,03	0,85
Não primeiro na ordem de nascimento	1,10 (0,66-1,84)	0,15	0,70	1,51 (0,79-2,89)	1,56	0,21

*Ajustado para sexo, idade e índice de massa corporal. IC95%: intervalo de confiança - IC95%; OR: *odds ratio*.

## DISCUSSÃO

Este é o primeiro estudo feito com uma amostra não clínica, composta de estudantes calouros de cursos de Ciências da Saúde com propósito de explorar a inter-relação entre fatores perinatais/neonatais e sintomas de TA. Já que alguns estudos indicaram que essa população é mais vulnerável ao desenvolvimento de TA,^(17,28)^ a presente investigação acrescenta dados relevantes ao conhecimento atual sobre os possíveis agentes envolvidos na etiologia desses transtornos. Assim, os principais achados são: (1) complicações obstétricas são positivamente associadas a sintomas de bulimia nervosa e (2) uma idade materna inferior a 25 anos no momento do parto é um fator de risco para sintomas de anorexia nervosa.

Nossos dados mostraram que os sintomas de bulimia nervosa são duas vezes maiores entre estudantes com complicações obstétricas do que entre seus pares que não as apresentaram. Em concordância com nossos resultados, Favaro et al.^(12)^ verificaram o mesmo padrão em mulheres com diagnóstico de anorexia nervosa. Ademais, Kelly et al.^(29)^ demonstraram que as complicações obstétricas podem estar envolvidas no início da esquizofrenia.

Já que o ambiente perinatal e o período inicial de desenvolvimento são capazes de produzir alterações de longa duração na estrutura cerebral e plasticidade em neonatos,^(30)^ assim como afetar a organogênese e o perfil epigenético em recém-nascidos,^(31)^ os possíveis mecanismos subjacentes aos efeitos deletérios de complicações obstétricas podem ser mediados por hipóxia, que causa dano ao desenvolvimento neurológico do recém-nascido.^(32)^ Além disso, a desnutrição durante a gravidez e no período pós-natal parece influenciar no controle central do gasto de energia e apetite na vida adulta.^(33)^ É importante notar que os diferentes tipos de complicações obstétricas têm implicações diversas para o risco de desenvolvimento de doença psiquiátrica.^(34)^

Um resultado surpreendente do presente estudo foi a menor incidência de sintomas de anorexia nervosa naqueles estudantes nascidos de mães com mais de 25 anos de idade; infelizmente, não há dados disponíveis a respeito desse tópico que nos ajudem a traçar hipóteses para essa questão. Porém esse fato intrigante deve ser investigado em futuros estudos para melhor elucidar essa possível associação.

Nosso estudo indica que o peso ao nascer, a amamentação, o tipo de parto e a ordem de nascimento não influenciam na probabilidade de sintomatologia de TA. Em concordância com nossos dados, Nicholls e Viner,^(35)^ em uma coorte nacional prospectiva de nascimentos, não identificaram relação de dose-resposta entre baixo peso ao nascer e risco de AN. Favaro et al.^(12)^ em um estudo longitudinal com coorte em uma população clínica com anorexia e bulimia nervosa não encontraram nenhuma associação com baixo peso de nascimento e ordem de nascimento como fator de risco para o desenvolvimento de TA.

O presente estudo tem algumas limitações que devem de ser consideradas. A medida autorrelatada de períodos perinatal e neonatal pode ter influenciado nos desfechos avaliados. Em função da vasta gama de complicações obstétricas relatadas no presente estudo, não fomos capazes de analisar a associação isolada de cada complicação obstétrica com os sintomas de TA. Por causa dessas limitações, a interpretação dos dados precisa ser feita com cautela. Dessa forma, futuros estudos realizados com maiores amostras, que analisem os fatores singulares e agrupados neonatais e perinatais podem elucidar e realçar nossa compreensão a respeito desse tópico relevante em Saúde Pública.

Os pontos fortes do presente estudo são as características dos voluntários: calouros de cursos de Ciências da Saúde, a inclusão de homens na amostra, e o ajuste da análise para sexo e IMC.

## CONCLUSÃO

Fatores perinatais e neonatais são importantes na patogênese de transtorno alimentar entre universitários. Da mesma forma, vigilância cuidadosa é necessária para evitar o aumento da incidência de transtorno alimentar nessa população. Sugerimos que as universidades prestem mais atenção a essa questão, ofereçam suporte psicológico aos alunos desde o início do curso; e deem informações sobre prevenção de transtorno alimentar durante as aulas iniciais para calouros de cursos de Ciências da Saúde, para evitar futuras complicações na próxima geração de profissionais de saúde. Mais estudos devem focar nos mecanismos subjacentes que correlacionam fatores neonatais aos transtornos alimentares.
